# Hereditary exostosis: An unexpected malignant transformation in a pediatric patient

**DOI:** 10.1016/j.radcr.2024.10.060

**Published:** 2024-11-13

**Authors:** Hadj Hsain Ihssan, Maslouhi Kaoutar, Lahlou Chaimae, Marrakchi Salma, Ezzaky Sara, Allali Nazik, Chat Latifa, El Haddad Siham

**Affiliations:** Radiology Department of HER, University Mohammed V, Rabat, Morocco

**Keywords:** Exostose, Hereditary, Bone

## Abstract

In this article, we report the case of an 8-year-old child with multiple hereditary exostoses, revealed by the rapid and painful growth of bone deformities in the right leg. After performing a CT scan, multiple exostoses were observed in the pelvic region and both lower limbs. Notably, the exostosis in the right fibula was larger and exhibited a thickened cartilaginous cap. Subsequent MRI confirmed the presence of signs of malignant transformation at this site. Although the literature suggests that malignant transformation is exceptional at this age, ongoing genetic studies may provide insight into the early occurrence of such complications.

## Introduction

Hereditary multiple exostosis, first described by John Hunter in 1786 and later by Boyer in 1814 [1,2], is an autosomal dominant heterogeneous disorder resulting from mutations in the EXT1, EXT2, and EXT3 genes located on chromosomes 8, 11, and 19 [3]. These genes play a role in chondrocyte differentiation. This disorder leads to the development of osteocartilaginous metaphyseal exostoses, primarily affecting long bones such as the femur, tibia, and humerus, with rare occurrences in the bones of the hands, feet, or ribs. The complications associated with the hereditary myltiples exostosis can be numerous and include bone deformities, restrictions of movement, nerve or vascular compression, and chronic pain. A relatively rare but serious complication is the malignant transformation of these exostoses into chondrosarcomas. first reported in 1886 [4,5], with subsequent documented cases in the literature, including 46 in Ochnser's investigation [6].

## Case report

A 8-year-old male child presented to the clinic with complaints of progressive pain in his left leg over the past several weeks. The pain was described as intermittent, exacerbated by physical activity, and causing difficulty in walking. The patient and his family noted no recent trauma or injury. The child had no previous significant medical history other than a family history of bone abnormalities, specifically exostoses, affecting several family members.

The family history was notable for multiple cases of exostoses in close relatives, including the patient's paternal uncle and cousin. Both were diagnosed with multiple hereditary exostoses (EHM). There was no prior history of malignant transformation in these cases, although the current presentation raised concern given the chronicity and severity of the pain. On clinical examination, the child was in good general condition, but a palpable irregular bony subcutaneous deformation was noted, causing discomfort upon palpation, there was no overlying skin erythema or signs of infection, the range of motion of the left knee was limited due to pain, neurological examination revealed no deficits, a thorough biological workup was conducted to assess general health, rule out other conditions, and evaluate any systemic impact. The following tests were performed: Hemoglobin (Hb): 11.8 g/dL (normal range: 11.5-15.5 g/dL for children, White Blood Cell (WBC) count: 6.5 × 10^9/L (normal range: 4.5-13.5 × 10^9/L), Platelet count: 250 × 10^9/L (normal range: 150-450 × 10^9/L), C-Reactive Protein (CRP): 2 mg/L (normal range: <10 mg/L), Calcium (Ca): 2.45 mmol/L (normal range: 2.2-2.7 mmol/L), Phosphorus (P): 1.35 mmol/L (normal range: 1.1-1.8 mmol/L).

To further evaluate the condition, a standard radiograph was performed, revealing multiple sessile bone formations, with the most concerning lesion located in the fibula, showing heterogeneous characteristics. A computed tomography (CT) scan of the pelvis and lower extremity was subsequently conducted, demonstrating exostoses in the pelvic bones and both lower extremities, some pedunculated and others sessile, all covered by a thin cartilaginous cap continuous with the underlying bone. However, the lesion in the right fibula exhibited increased size, irregular borders, thickened cartilaginous cap, and heterogeneous bone appearance with contained calcifications,. Magnetic resonance imaging (MRI) was performed for further characterization, revealing an osseous outgrowth in the right fibular metaphysis, covered by a moderate thick cartilaginous cap measuring 6m m and enhanced after gadolinium injection. The underlying bone showed heterogeneous characteristics in hetereogne signal T2, and soft tissue infiltration was observed, malignant degenerescne was highly suspected, and a bone biopsy was proposed and carried out by the surgeon, histological study revealed mesenchymal tumor proliferation made up of lobukes separated by fibrous tissue, spindle cells with irregular nuclei sometimes in duplicate with moderate cytonuclear atypia.

Given the malignant transformation of the exostosis, the case was referred to a multidisciplinary team involving orthopedic oncologists and pediatric surgeons. The following management steps has been programmed: a complete excision of the tumor with clear margins was performed to prevent recurrence or metastasis, with good postoperative recovery, and physical therapy was implemented after surgery to restore mobility and function to the left leg, the patient is currently undergoing a period of recovery, and continuous monitoring with regular imaging is planned to assess the development of new exostoses or any signs of malignant transformation elsewhere.

In view of the fairly early appearance of a malignant transformation of the exostosis, which is unexpected at this age, a genetic study in search of a possible mutation was proposed to the family, as well as a broad screening of the various members of the family, but this has not been carried out to date for financial reasons.

## Discussion

Hereditary multiple exostosis, also known as Bassel-Hagen disease in its multiple forms, has been classified by the World Health Organization (WHO) as a benign condition characterized by both cartilaginous and bony components [7]. generally occurs between the ages of 2 and 10. This period coincides with the phases of rapid growth of the long bones, particularly during childhood and puberty, It's a fairly a rare condition, Schmale et al. conducted a study looked at the frequency of inherited exostosis in 305 patients in the United States. According to their estimates, there is 1 case of the disease per 50,000 to 100,000 people, with an estimated age of onset of 12 years [8], while another study conducted by Francannet et al. in France on 73 families with EHM showed the same prevalence as the previous one, with a more precose age this time estimated at 10 years. They also observed that mutations in the EXT1 gene often lead to more severe clinical expression than those linked to EXT2, with a greater frequency of complications [9], meanwhile a comprehensive investigation into the genetic alterations linked to multiple exostosis was conducted by Wuyts et al. Since these genes code for enzymes necessary for the manufacture of bone proteoglycans, they found that mutations in the EXT1 and EXT2 genes account for most cases of hereditary exostosis. A deeper comprehension of the disease's molecular pathogenesis has been made possible by this work [10].

**Fig. 1:**
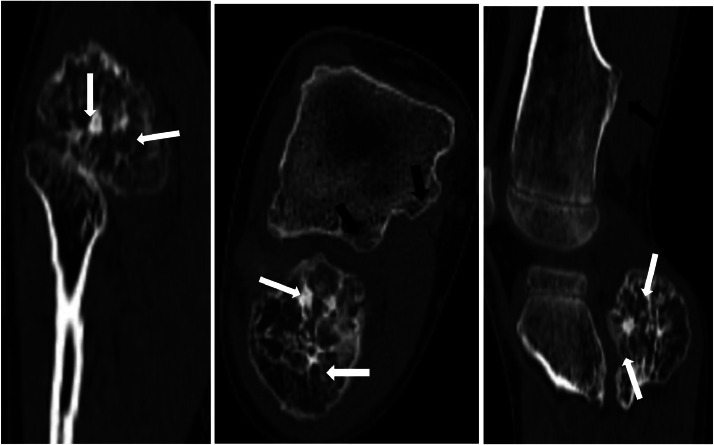
CT coronal, axial and sagittal slices show a heterogeneous fibular bone outgrowth with irregular contours and calcifications (whites arrows), and other homogeneous tibial bone outgrowths with the same structure as the adjacent bone (blacks arrows).

**Fig. 2:**
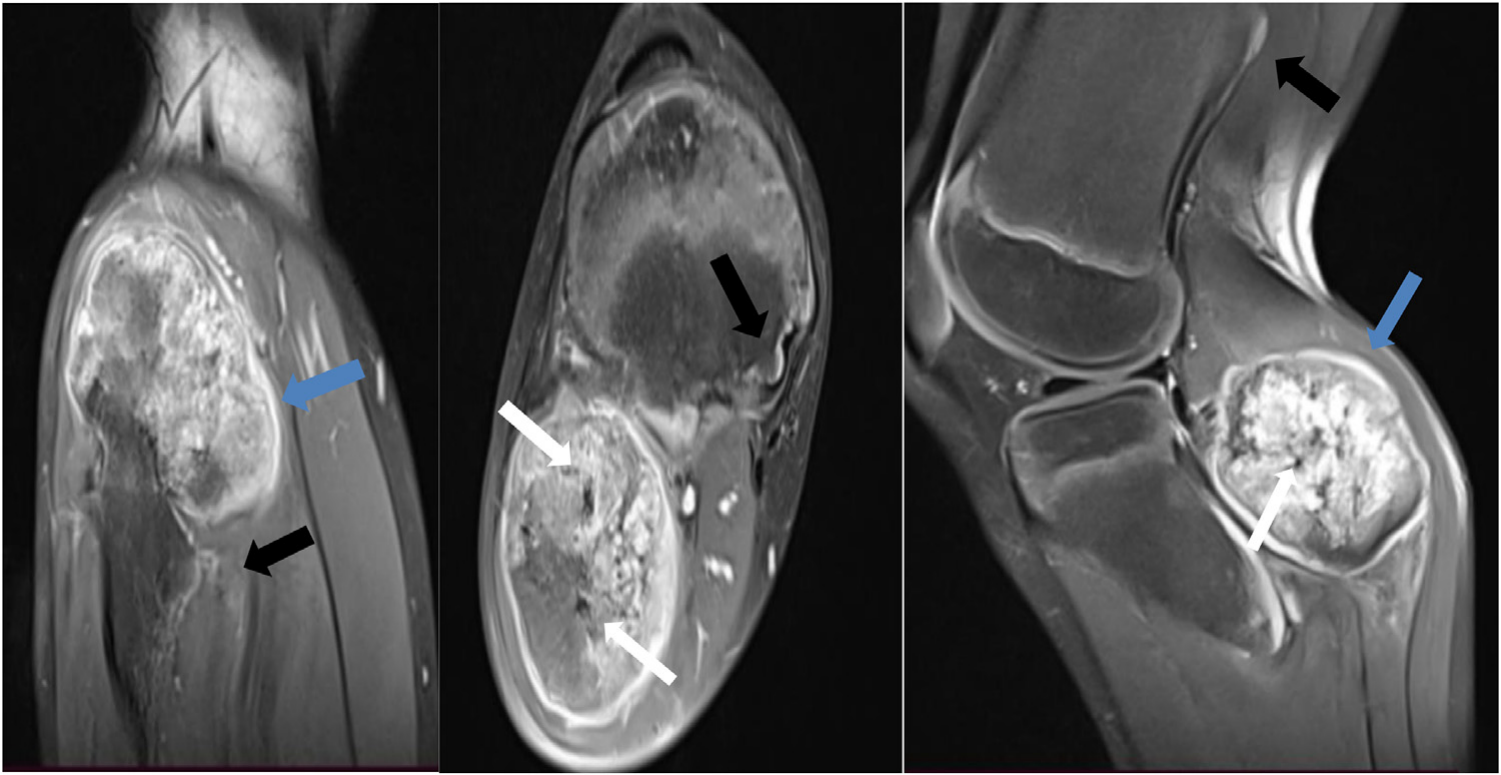
Axial and sagittal coronal sections in T1 sequence with fat saturation, showing a fibular exostosis with heterogeneous signal, containing areas of asignal (white arrows) surrounded by a cartilaginous cuff with hyper signal(blue arrows), with other tibial outgrowths in isosignal (black arrows).

**Fig. 3:**
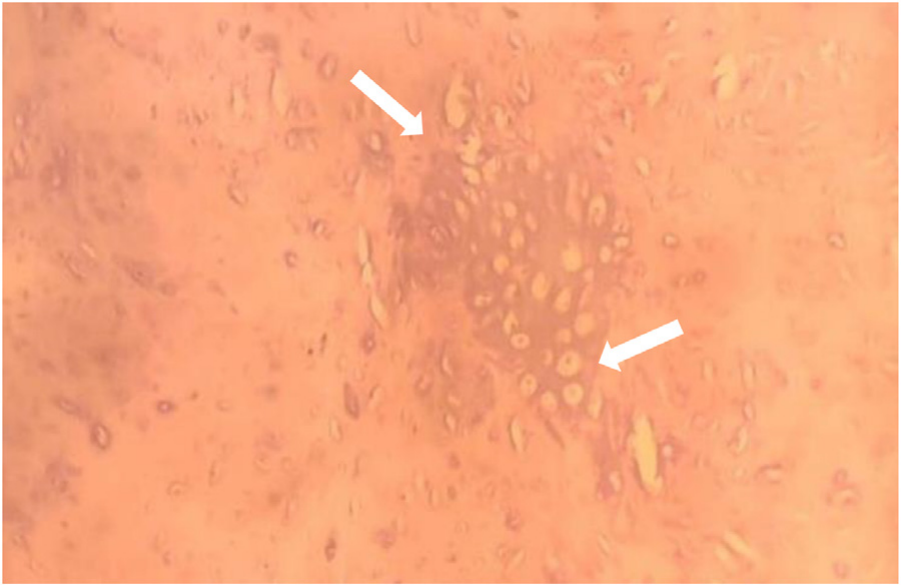
With HES coloration: mesenchymal tumor proliferation made up of lobukes separated by fibrous tissue, spindle cells with irregular nuclei (arrows)sometimes in duplicate with moderate cytonuclear atypia.

Clinically, this pathology remains silent and only becomes evident in the presence of complications associated with hereditary multiple exostosis include nerve compression, tendon entrapment, and vascular compression. Additionally, rare complications have been reported in the literature, such as hemothorax secondary to an exostosis, with approximately thirty cases documented in the last 2 decades [11]. Cases of multiple exostoses with digestive symptoms have also been described. For instance, Barros Filho et al. reported a case of a 16-year-old adolescent with dysphagia secondary to an exostosis of the anterior arch of the atlas [12]. Two cases of digestive obstruction secondary to exostoses were documented in a 16-year-old adolescent [13] and a 31-year-old man [14]. However, malignant transformation remains the most fearsome complication, first reported in 1886 [5], has been documented in 50 cases in the literature, including 46 cases included in Ochnser's investigation [6]. The reported incidence of malignant transformation in exostoses varies widely, ranging from 3% to 25%. Chondrosarcoma has been observed in 3 out of 28 patients studied by Hennekam [15]. However, it's important to note that only family members evaluated at the hospital were included in this estimation, and the verification was not comprehensive. Based on an examination of 272 cases of multiple exostoses treated at the Mayo Clinic, Dahlin estimated the incidence of chondrosarcoma to be approximately 10% [16]. A French study of 175 patients with hereditary multiple exostosis, reported by Legeai-Mallet et al., estimated the frequency to be between 0.6% and 2.8% [17]. Generally, malignant transformation occurs between the ages of 19 and 39, in contrast to osteochondromas, which have a higher incidence at the age of 45 ,whereas our patient showed signs of malignant transformation very precociously at the age of 6, which makes our case rather unusual, thus motivating a detailed genetic study in search of a new mutation, but unfortunately, due to lack of material, it was not carried out (Fig. 1, Fig. 2, Fig. 3).

In 90% of cases, malignant transformation leads to chondrosarcoma, characterized by low malignancy rates [18]. Evolution into undifferentiated chondrosarcoma has also been described, with a less favorable prognosis [16]. The most frequent sites of malignant transformation are the scapula, proximal humerus, pelvis, proximal femur, followed by the distal femur, proximal or distal leg, and forearm. Both proximal and distal parts of the arm, as well as the hand, have been reported as sites of transformation [13]. Clinically, exostoses are typically painless, and the development of pain or rapid growth is indicative of malignancy, which was the case with my patient, where leg pain was a reason for consultation.

Radiologically, exostoses are generally benign tumors, often sessile or pedunculated, covered by a carti- laginous cap that is continuous with the cartilage of the underlying bone. This cap is usually thin, with an average thickness of 6 to 8 mm, but it can reach up to 15 mm during growth [18]. Malignant transformation occurs within the cartilaginous cap [19]. The initial signs of transformation should be detected through ultrasound or MRI. A cartilaginous cap measuring less than 1 cm or 2 cm, as well as a lobulated appearance, strongly suggests chondrosarcomatous transformation. Other radiological signs that should raise suspicion include degeneration, such as the presence of irregular and heterogeneous calcifications extending beyond the limits of the exostoses, or the presence of soft tissue masses surrounding an exostosis. The risk of degeneration increases with size (greater than 10 cm) and is associated with abundant myxoid substance, cyst formation, and local aggressiveness [19], in our case, in view of the atypical appearance of the exostosis protruding from the right fibular joint, which appeared heterogeneous and contained calcifications, we carried out an MRI scan, which showed a cartilaginous cuff 6mm thick with a heterogeneous T2 signal and asignalled areas on all sequences related to calcification, malignant degenerescne was highly suspected, and a bone biopsy was proposed and carried out by the surgeon.

Bone scintigraphy can assist in identifying active osteochondromes, which exhibit intense radiolabeled bone uptake. However, it does not differentiate between benign osteochondromas and malignant transformation [10]. In fact, there is no absolute norm, and all radiologically suspicious exostoses should be surgically excised. A pathological examination of the entire site provides clear diagnostic evidence, our patient was scheduled for surgical removal of suspected fibular exostosis with monitoring of other growths.

Histologically, chondrosarcomas developing from exostoses show signs of malignancy, such as hypercellularity, marked pleiomorphism and disorganized proliferation of cartilage cells. In contrast to the normal cartilage present in benign exostoses, abnormal mitoses may be observed, as well as necrosis and areas of dystrophic calcification, which are less common in benign exostoses. Increased cellularity and invasion of surrounding tissues, including underlying bone and adjacent structures, are indicators of malignancy [20].

## Conclusion

Malignant degeneration remains a formidable complication with a significant incidence. Therefore, patients with hereditary exostotic disease and their affected families must be informed about the substantial risk of chondrosarcoma during genetic counseling and orthopedic evaluation, early detection of this transformation is crucial to improving prognosis, as regular clinical monitoring can prevent or treat serious complications. Thus, despite the benign nature of the majority of exostoses, rigorous management and careful follow-up of patients with EHM are essential, particularly to monitor the risk of malignant transformation and intervene promptly should the need arise.

Consequently, it is advisable for them to undergo an annual clinical follow-up and radiological screening, and potentially additional diagnostic examinations, including periodic MRI scans.

## Patient consent

For the case report written informed consent for publication of the case was obtained by the first author of the case report.
